# Lytic Promoters Express Protein during Herpes Simplex Virus Latency

**DOI:** 10.1371/journal.ppat.1005729

**Published:** 2016-06-27

**Authors:** Tiffany A. Russell, David C. Tscharke

**Affiliations:** John Curtin School of Medical Research, The Australian National University, Canberra, Australian Capital Territory, Australia; Geisel School of Medicine at Dartmouth, UNITED STATES

## Abstract

Herpes simplex virus (HSV) has provided the prototype for viral latency with previously well-defined acute or lytic and latent phases. More recently, the deep quiescence of HSV latency has been questioned with evidence that lytic genes can be transcribed in this state. However, to date the only evidence that these transcripts might be translated has come from immunological studies that show activated T cells persist in the nervous system during latency. Here we use a highly sensitive Cre-marking model to show that lytic and latent phases are less clearly defined in two significant ways. First, around half of the HSV spread leading to latently infected sites occurred beyond the initial acute infection and second, we show direct evidence that lytic promoters can drive protein expression during latency.

## Author Summary

Herpes simplex virus, which causes cold sores and genital herpes, has active and inactive (or latent) phases of infection that have been considered to be distinct. In this study we found that the active phase of infection, including spread in the nervous system, continues longer than has been previously appreciated. We also show evidence that virus genes previously only associated with active infection are turned on during latency. These genes are of particular interest because other work has found that they are targets of the immune response to HSV. The extent and nature of residual viral activity during latency is important to understand because it may suggest therapeutic targets to reduce recurrent HSV disease.

## Introduction

The herpesviruses are a large family of viruses the members of which share the ability to persist in their host, typically using latency and reactivation as a survival strategy. Herpes simplex virus type I (HSV-1) is the best characterized herpesvirus and the poster-child for classically defined viral latency. HSV-1 enters the body through the skin or mucosal surfaces where it initiates a productive primary infection that rapidly spreads to innervating sensory neurons. This productive, or lytic phase of infection proceeds via the expression of roughly 80 viral functions in an ordered cascade comprising immediate early (IE), early (E) and then late (L) genes [1]. After the primary lytic phase is halted by the development of an adaptive immune response, a latent reservoir of virus remains in some sensory neurons from which it may reactivate periodically causing renewed episodes of productive infection [2].

An operational definition of HSV-1 latency is the persistence of viral DNA in the absence of infectious virus. In mouse models, which have been a mainstay of HSV-1 research, latency has been found to be especially profound, with reactivation very difficult to induce *in vivo* [3–6]. Historically, viral transcription during latency was considered to be restricted to a family of RNA transcripts, the latency associated transcripts (LATs) [7–9]. This view is consistent with repressive marks being found on chromatin associated with the majority of the viral genome during latent infection [10, 11]. However, lytic gene transcription in latency was noted in sporadic, but persistent reports over many years [12–22]. In addition, repression of the genome is not absolute and transcription from the LAT region may play a role in maintaining this state [23]. A recent single cell analysis of transcription in latently infected mouse dorsal root ganglia (DRG) found that while levels were low, lytic gene transcripts were found in more than two thirds of all latently-infected neurons [24]. Further, host antiviral and survival gene transcription was modulated in response to the presence of lytic viral transcripts, suggesting biologically relevance [24].

Direct evidence for translation of lytic transcripts has been harder to find. However, the retention of activated, virus-specific CD8^+^ T cells within the sensory ganglia of latently infected individuals provides indirect evidence for the translation of at least some lytic proteins [25–28]. In the commonly used C57Bl/6 mouse model, more than half of these anti-HSV CD8^+^ T cells recognize an epitope derived from the late protein, glycoprotein B (gB; gB_498_) [25, 29] but other lytic gene products, including ICP6 are also targets [29].

Cre-reporter mice, such as ROSA26R, coupled with viruses that express Cre recombinase provide a highly sensitive model to detect transient and/or low levels of viral protein production [24, 30–32]. In this model, Cre can be expressed under various HSV-1 promoters leading to the permanent marking of infected cells in ROSA26R mice by catalyzing a recombination that allows expression of *LacZ* from the genome of that cell. To date, these studies using Cre-reporter models have not revealed evidence of lytic gene expression during latency, contrary to the immunological studies noted above [30, 31]. However as yet, no promoter that drives a major target of anti-HSV CD8^+^ T cells has been tested. To bridge the gap between the immunological-based studies and those that utilize Cre-reporter models, we constructed and tested a set of HSV-1 viruses that express Cre under promoters that drive well-characterized CD8^+^ T cell targets or proteins with an immune evasion function [29, 33–37]. We found that unlike promoters tested previously, those driving gB and ICP6 can produce protein during HSV latency.

## Results and Discussion

### Characterization of HSV-1 recombinants expressing Cre *in vitro* and *in vivo*

**Fig 1:**
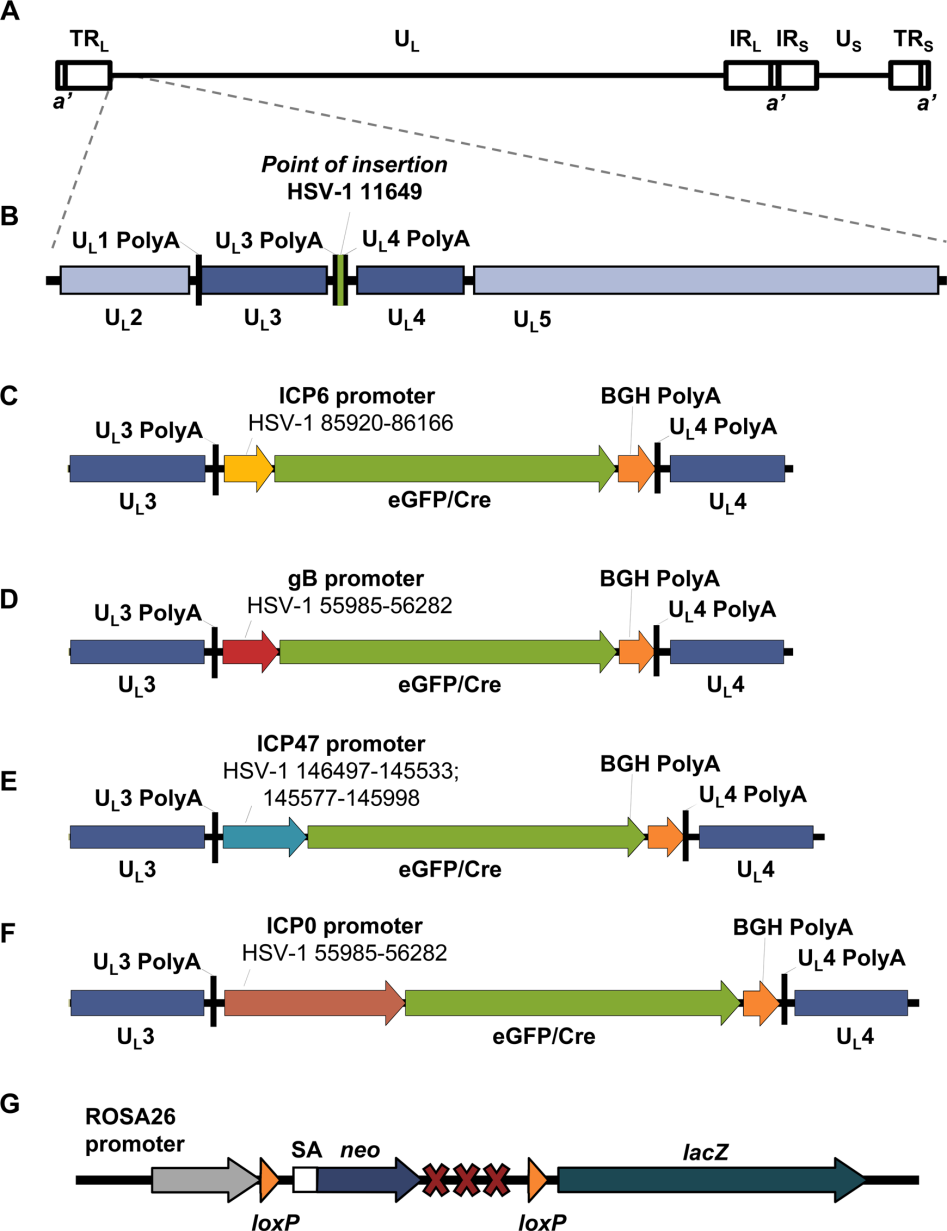
Design of HSV-1 viruses that express eGFP/Cre. (A) Schematic representation of the HSV-1 genome (to scale). (B) Schematic representation of the U_L_3/U_L_4 intergenic region, with the position at which the *eGFP/Cre* cassette is inserted indicated in green, and the base pair position indicated using coordinates from the HSV-1 KOS genome (JQ673480). Schematic representation of the *eGFP/Cre* gene under the control of the (C) ICP6 promoter, (D) gB promoter, (E) ICP47 promoter or (F) ICP0 promoter inserted into the HSV-1 genome, with the sequences of the promoter indicated based on HSV-1 KOS genome coordinates. (G) Schematic representation of the ROSA26 locus of ROSA26 mice. A splice acceptor sequence (SA) is located upstream of a neomycin gene (*neo*) followed by a triple polyadenylation site, all of which is flanked by *loxP* sites. The ROSA26 promoter is located upstream of this transgene, while the *lacZ* gene is located downstream. Following Cre-mediated recombination, the *neo* transgene is removed and *lacZ* is constitutively expressed from the ROSA26 promoter.

Four recombinant viruses were constructed for use in which an *eGFP/Cre* fusion gene was introduced into the U_L_3/U_L_4 intergenic region of HSV-1 KOS under the control of the IE R_S_1 (ICP0) or U_S_12 (ICP47), E U_L_39 (ICP6) or L U_L_27 (gB) promoters, respectively (Fig 1). HSV-1 pC_eGC, in which the *eGFP/Cre* fusion gene is expressed from the cytomegalovirus immediate early (CMV IE) promoter, has been described previously [38]. Fidelity to the expected kinetic class was confirmed *in vitro* using acyclovir and cycloheximide, as described by Summers and Lieb [39]. Only promoters for ICP0 or ICP47 were active in the presence of cycloheximide, consistent with them being of the IE class, but as expected all were active in cultures treated with acyclovir (S1A and S1C Fig). Further all recombinant viruses replicated with parental kinetics in Vero cells *in vitro* (S2A–S2E Fig) and in the skin and innervating dorsal root ganglia (DRG) of tattoo-infected C57Bl/6 mice (S2F–S2H Fig). Finally, all viruses were able to be reactivated from the DRG of latently infected mice by explant culture. Therefore, all these recombinant viruses are phenotypically similar to parental HSV-1 KOS [38, 40]. As a final control in our experimental model we formally demonstrated that β-gal expression only occurs in ROSA26R mice during HSV-1 infection when Cre is present (S3 Fig).

### HSV spread continues beyond the peak of acute infection

**Fig 2:**
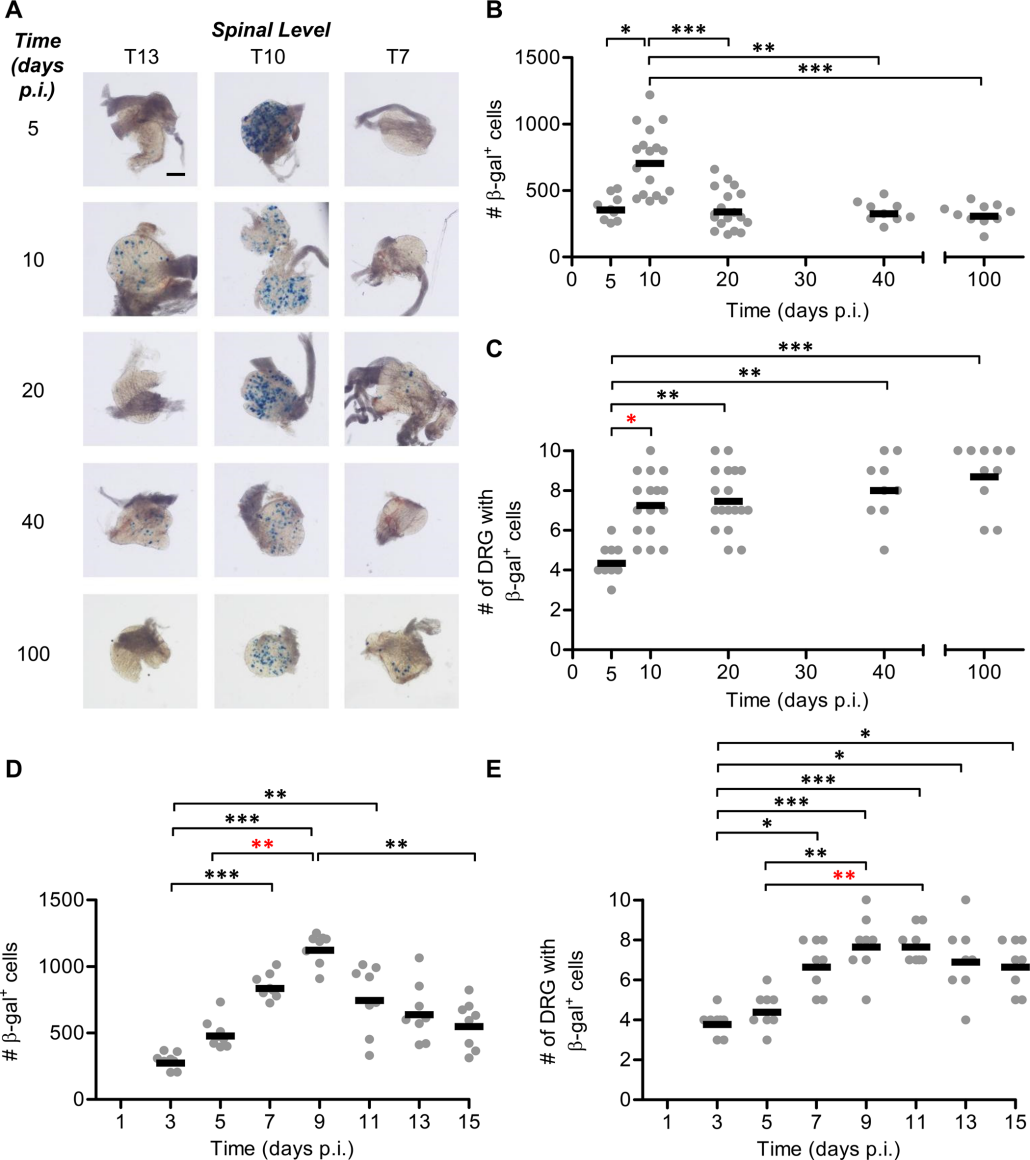
HSV continues to spread beyond the acute infection before latency is stably established. Groups of ROSA26 mice were infected with 1 × 10^8^ PFU/mL HSV-1 pC_eGC by tattoo on the flank and were culled at indicated times p.i. The innervating DRG were removed (from spinal levels L1 to T5) and the total number of β-gal^+^ cells per mouse was determined. (A) Representative photomicrographs of DRG at spinal levels T13, T10 and T7 of a single mouse for each day p.i. taken at a 40× magnification (scale bar = 300 μm, as indicated on top left image). (B) Total number of β-gal^+^ cells per mouse (data from some mice have been published previously [24]) and (C) spread of virus as indicated by the number of DRG containing β-gal^+^ cells. Each point represents a single mouse and the bar represents the mean cell count (*n* = 9–18 per day p.i.). The results of four independent experiments were pooled. (D) Total number of β-gal^+^ cells per mouse and (E) spread of virus as indicated by the number of DRG containing β-gal^+^ cells, as measured at two day intervals between days 3 and 15 p.i. Data from two independent experiments were pooled. (*p < 0.05, **p < 0.01, ***p < 0.001; red denotes differences of particular note).

**Fig 3:**
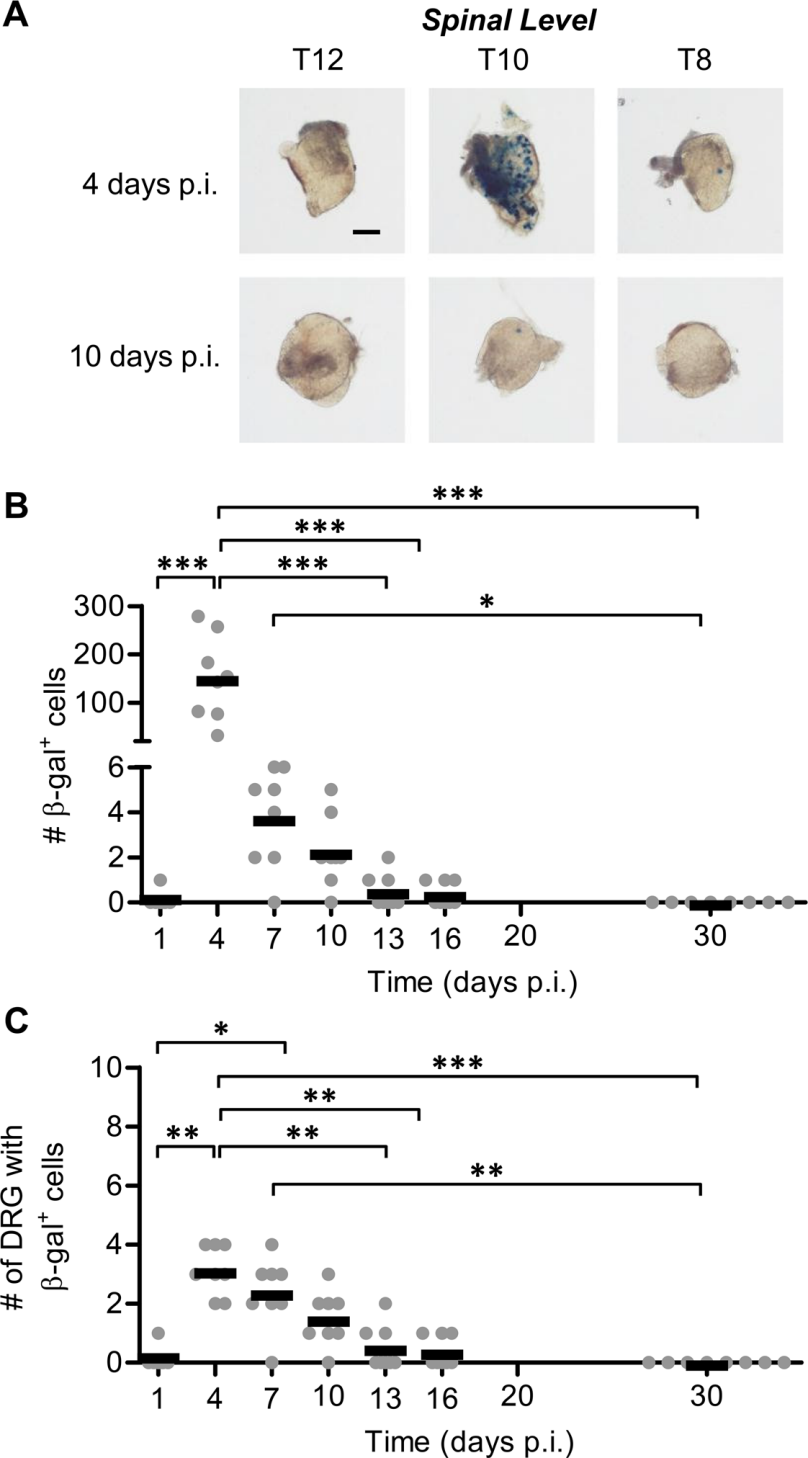
Conventional reporter genes show only minor lytic gene expression after the peak of acute infection and none in latency. Groups of C57Bl/6 mice were infected with KOS6.β by tattoo on the flank and were culled at the indicated times p.i. for the determination of the number of β-gal^+^ cells per DRG. (A) Representative photomicrographs of DRG at spinal levels T12, T10 and T8 of a single mouse for either 4 or 10 days p.i. taken at 40× magnification (scale bar = 300 μm, as indicated on the top left image). (B) The total number of β-gal^+^ cells per mouse was estimated. (C) The spread of virus as determined by the number of DRG which contain at least one β-gal^+^ cell. The results of two independent experiments are pooled (*n* = 8), with each point representing a single mouse and the bar representing the mean cell count. (**p < 0.01, ***p < 0.001).

Before investigating lytic promoter activity during latency, we used the increased sensitivity of the Cre-marking model to define more closely the time period over which HSV-1 spreads to sites where latency is established. These experiments used a previously described virus, namely HSV-1 pC_eGC, that has not been seen to mark neurons in latency, but from which there is a brief expression of Cre in every infected cell irrespective of whether an acute or latent infection ensues [24, 30]. Tattoo infection of the flank was used as this allows the spread of HSV-1 in the peripheral nervous system to be monitored by examining multiple DRG along the spine with respect to the infected dermatome [38, 41, 42]. HSV-1 pC_eGC continued to mark many new neurons between day 5 and day 10, which is beyond the peak of infection as demonstrated by other methods (Fig 2A and 2B) [42, 43]. Further, we were surprised to find that HSV-1 continued to spread through the nervous system, reaching new DRG after day 5 (Fig 2C). After day 10 the number of marked neurons, but not infected DRG, fell (presumably due to the death of some neurons) until day 20, but remained stable thereafter. A follow up experiment tracking marked neurons at two day intervals showed a peak in both the total number of β-gal^+^ cells and infected DRG at 9 days p.i. (Fig 2D and 2E). To confirm kinetics of lytic viral gene expression by a more conventional method in our flank tattoo model, mice were infected with HSV-1 KOS.6β, a virus that expresses β-gal under the control of the early U_L_39 (ICP6) promoter [39]. The expected pattern of HSV-1 acute infection was found using this method, with a peak in β-gal expression at 4 days p.i. and a rapid decline thereafter, with few marked neurons detected after day 7 (Fig 3A and 3B). The total number of DRG seen to be infected at any one time followed the same pattern as total number of marked neurons, but notably was never as wide as revealed by the Cre marking experiments (Fig 3C). We did observe one or two marked neurons in a minority of mice as late as day 16, but none at day 30, consistent with other reports of sporadic viral activity during the establishment phase of latency [44–49]. We suggest that the low level of detectable gene expression can be reconciled with the peak of Cre marking at day 10 if the death of infected neurons is taken into account. At the peak of the acute infection, the infection of neurons may be matched by the death and therefore loss of neurons, masking the spread of virus. At later times, when immune responses, such as CD8^+^ T cells, are active and having a protective effect [50–52], marking by Cre peaks as spread and survival outpaces neuronal death. Importantly, as revealed by these experiments, this unexpected late virus spread increases the pool of latently infected neurons. We suggest that the establishment phase is a time of balance between non-destructive immune control and virus spread. Further, the extent of the spread at this time might help explain why the repressive chromatin marking of HSV genomes associated with latency continues for around two weeks after infection [11].

Finally, the stability of numbers of neurons marked by this virus over the period of latency confirms previous reports and suggests that there is no significant full reactivation of virus and spread beyond the establishment phase. The only way that reactivation and spread could be consistent with these data is if each reactivating neuron was lost and then replaced by an average of one newly infected neuron. While this remains a possibility, it is a less simple explanation than latency being stable in our model.

### ICP0 promoter expression detected during late HSV-1 spread, but not in latency

**Fig 4:**
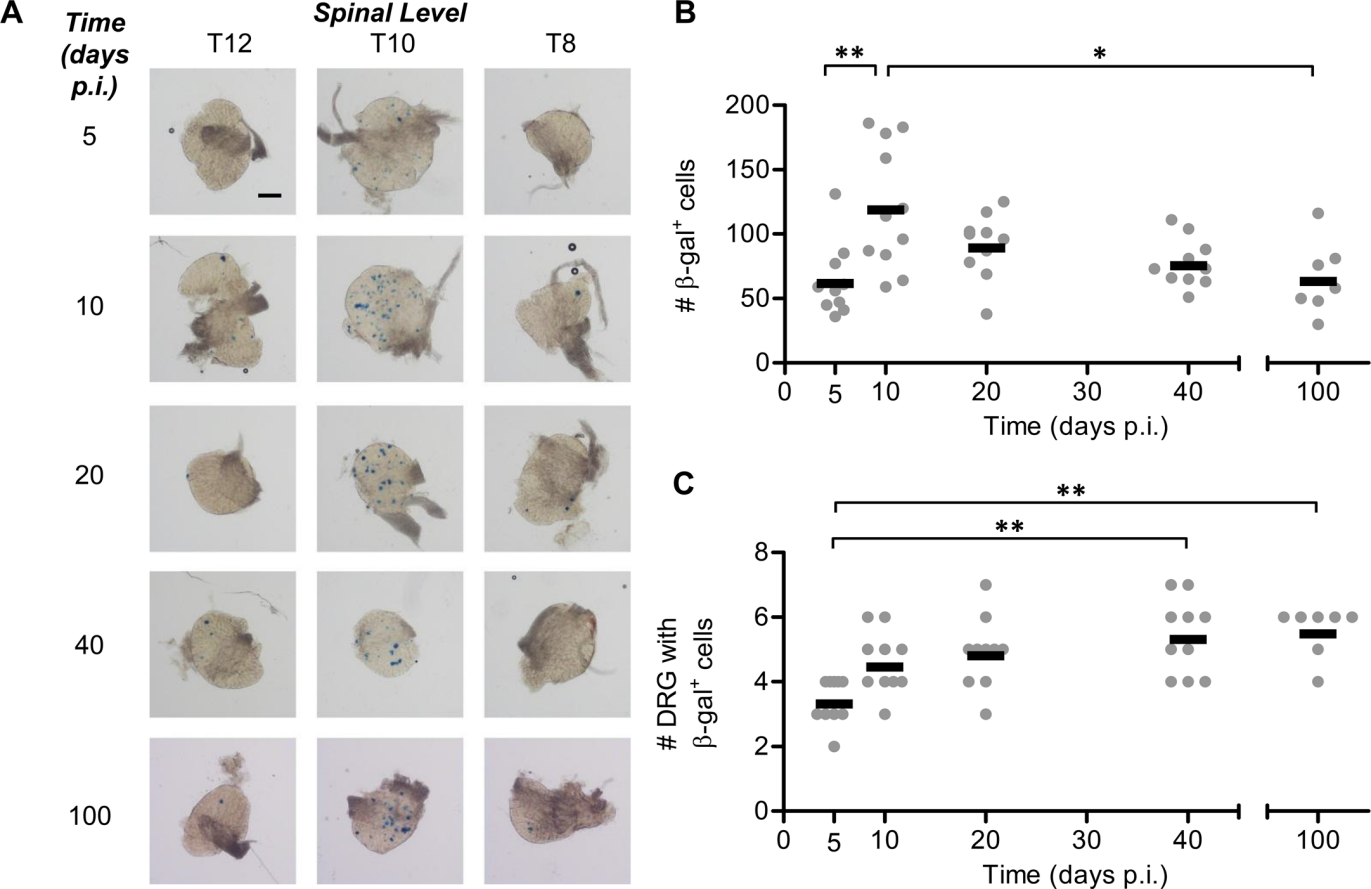
The promoter for ICP0 is active through the establishment phase but not true latency. Groups of ROSA26 mice were infected with HSV-1 pICP0_eGC by tattoo on the flank and were culled at the indicated times p.i. and the number of β-gal^+^ cells in each DRG was determined. (A) Representative photomicrographs of DRG at spinal levels T12, T10 and T8 of a single mouse for each day p.i. taken at 40× magnification (scale bar = 300 μm, as indicated on top left image). (B) The total number of β-gal^+^ cells per mouse and (C) the spread of virus as indicated by the number of DRG containing β-gal^+^ cells was determined. The data were pooled from three independent experiments (*n* = 10–11 for each time point). (*p < 0.05, **p < 0.01)

Next we used a recombinant HSV-1 that expressed Cre under the control of the ICP0 promoter. When used to infect ROSA26R mice, this virus led to the marking of neurons over time in a relative kinetic pattern very similar to that seen above when Cre was driven by the ectopic CMV-IE promoter (Fig 4). This indicates that in some cases the virus that spreads after the first week of infection is initiating lytic gene expression. Notably, no further marking was noted during latency, which matches published results for the ICP0 promoter [30]. The original interpretation of this finding was that the ICP0 promoter does not lead to protein expression during HSV latency [29]. However, it remains possible that the ICP0 promoter is engaged, but only in those neurons that have survived previous expression from this promoter (and so in our experiment were already marked). A second possibility is that protein expression might lead to full reactivation and death of the neuron, but this would need to be balanced by spread to a similar number of neurons that are lost, which seems unlikely. Finally, Cre expression from the ICP0 promoter in latency might be too low or brief to catalyze recombination of the loxP sites in ROSA26R mice. Despite these caveats, we suggest that lack of protein production from this promoter during latency remains the least complicated and therefore most likely explanation.

### HSV-1 lytic promoters associated with T cell recognition generate protein during latency

**Fig 5:**
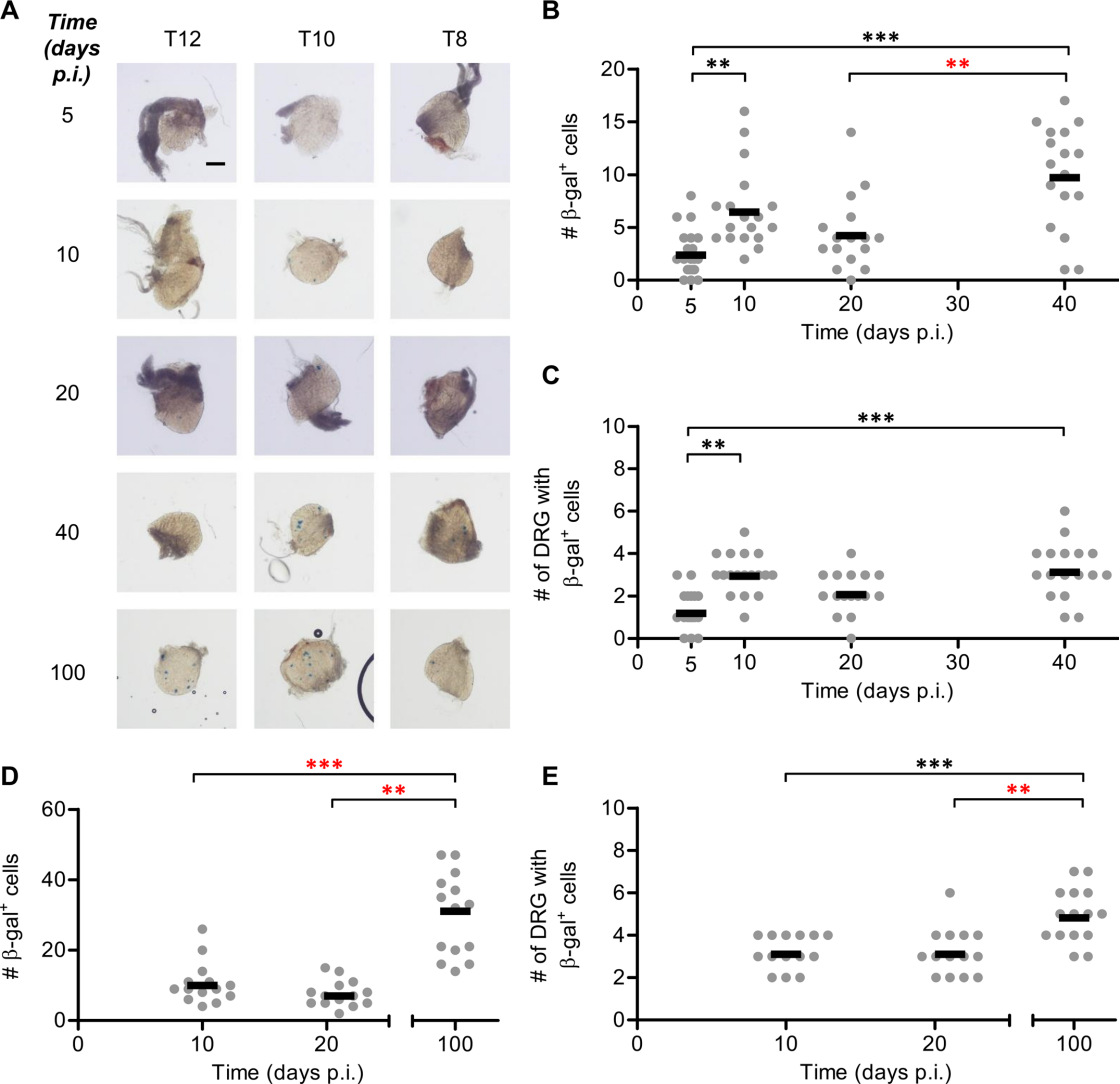
The promoter for gB drives protein expression during latency. Groups of ROSA26 mice were infected with HSV-1 pgB_eGC by tattoo on the flank and were culled at either (B, C) 5, 10, 20 and 40, or (D, E) 10, 21 and 100 days p.i. for determination of the number of β-gal^+^ cells per DRG. (A) Representative photomicrographs of DRG at spinal levels T12, T10, and T8 of a single mouse for each day p.i. taken at a 40× magnification (scale bar = 300 μm, as indicated on top left image). To examine the accumulation of β-gal^+^ cells throughout the acute infection and establishment of latency, (B) the total number of β-gal^+^ cells per mouse and (C) the spread of virus as indicated by the number of DRG containing β-gal^+^ cells was determined. The data are pooled from 5 independent experiments, with each point represents a single mouse and the bar represents the mean cell count (*n* = 16–18 per day p.i.). To examine the accumulation of β-gal marked cells in ROSA26 mice infected with HSV-1 pgB_eGC over the long term the (D) total number of β-gal^+^ cells per mouse and (E) the spread of virus as indicated by the number of DRG containing at least one β-gal^+^ cell is shown. The data are pooled from 3 independent experiments (*n* = 14–15). (**p < 0.01, ***p < 0.001; red denotes differences of particular note).

**Fig 6:**
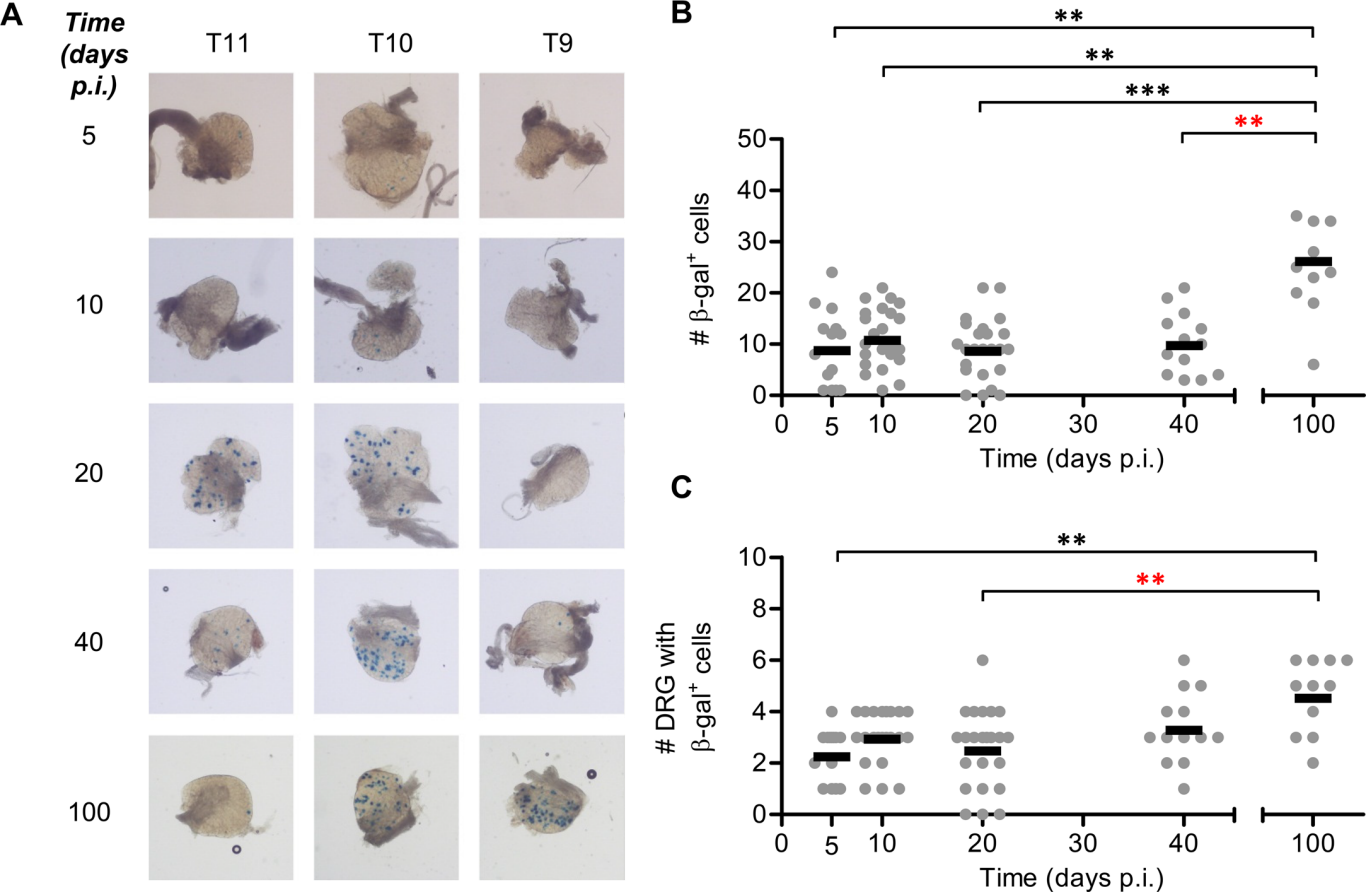
The promoter for ICP6 drives protein expression during latency. Groups of ROSA26 mice were infected with HSV-1 pICP6_eGC by tattoo on the flank and were culled at 5, 10, 20 and 40, or 10, 20, and 100 days p.i. for determination of the number of β-gal^+^ cells per DRG. (A) Representative photomicrographs of DRG at spinal levels T11, T10, and T9 of a single mouse for each day p.i. taken at 40× magnification (scale bar = 300 μm, as indicated on top left image). (B) The total number of β-gal^+^ cells per mouse and (C) the spread of virus as indicated by the number of DRG containing β-gal^+^ cells was determined. The data were pooled from six independent experiments, with each point representing a single mouse and the bar representing the mean cell count (*n* = 11–24 per day p.i.). Differences between groups were assessed using Kruskal Wallis test with Dunn’s posttest for pairwise comparison (**p < 0.01, ***p < 0.001; red denotes differences of particular note).

ICP0 is not a target of CD8^+^ T cells in mice [53] therefore, we tested two further viruses: These expressed Cre under the control of the promoters for gB and ICP6, the best established targets of anti-HSV CD8^+^ T cells in C57Bl/6 mice [34, 36]. When ROSA26R mice were infected with a HSV-1 expressing Cre from the gB promoter, marking of new neurons was seen between days 5 and 10 as now expected. However with this virus, a second significant increase in marked neurons was seen between days 20 and 40 consistent with expression of this promoter during latency (Fig 5A and 5B). The number of DRG with marked neurons was higher at day 40 than day 5, but not compared with days 10 or 20 (Fig 5C). To verify and extend the times examined, further experiments were done and the continued marking of neurons was observed again, this time between days 21 and 100 (Fig 5D). There was also a significant increase in the number of DRG with marked neurons at day 100, compared with day 10 (Fig 5E). These are most likely neurons that proceeded to latent infection without prior gB expression, but then initiated gB expression for the first time during latency because our experiments with HSV-1 pC_eGC show that HSV spread is complete by day 9 or 10 (Fig 2). Further support that some lytic promoters can drive protein expression during latency was then obtained using an HSV-1 expressing Cre from the ICP6 promoter. Similar to the gB promoter experiments above, neurons continued to be marked during latency and also more DRG contained marked neurons at day 100 compared with day 20 (Fig 6). The expression of β-gal in ROSA26R mice is dependent on the presence of functional Cre protein, implying that the gB and ICP6 promoters are driving HSV-1 protein production during latency. Further, the accumulation of marked neurons indicates that these cells survive this lytic gene expression. This scenario is incompatible with the concept that viral protein expression during latency heralds full reactivation leading to the death of the neuron [12, 45, 54]. It is more consistent with either a) limited stochastic lytic gene expression being an integral part of latency [24] or b) the early presentation of these proteins to CD8^+^ T cells providing an opportunity for reactivation to be halted at a very early stage by an immune mechanism [50, 51].

### The ICP47 promoter generates protein during establishment of latency

**Fig 7:**
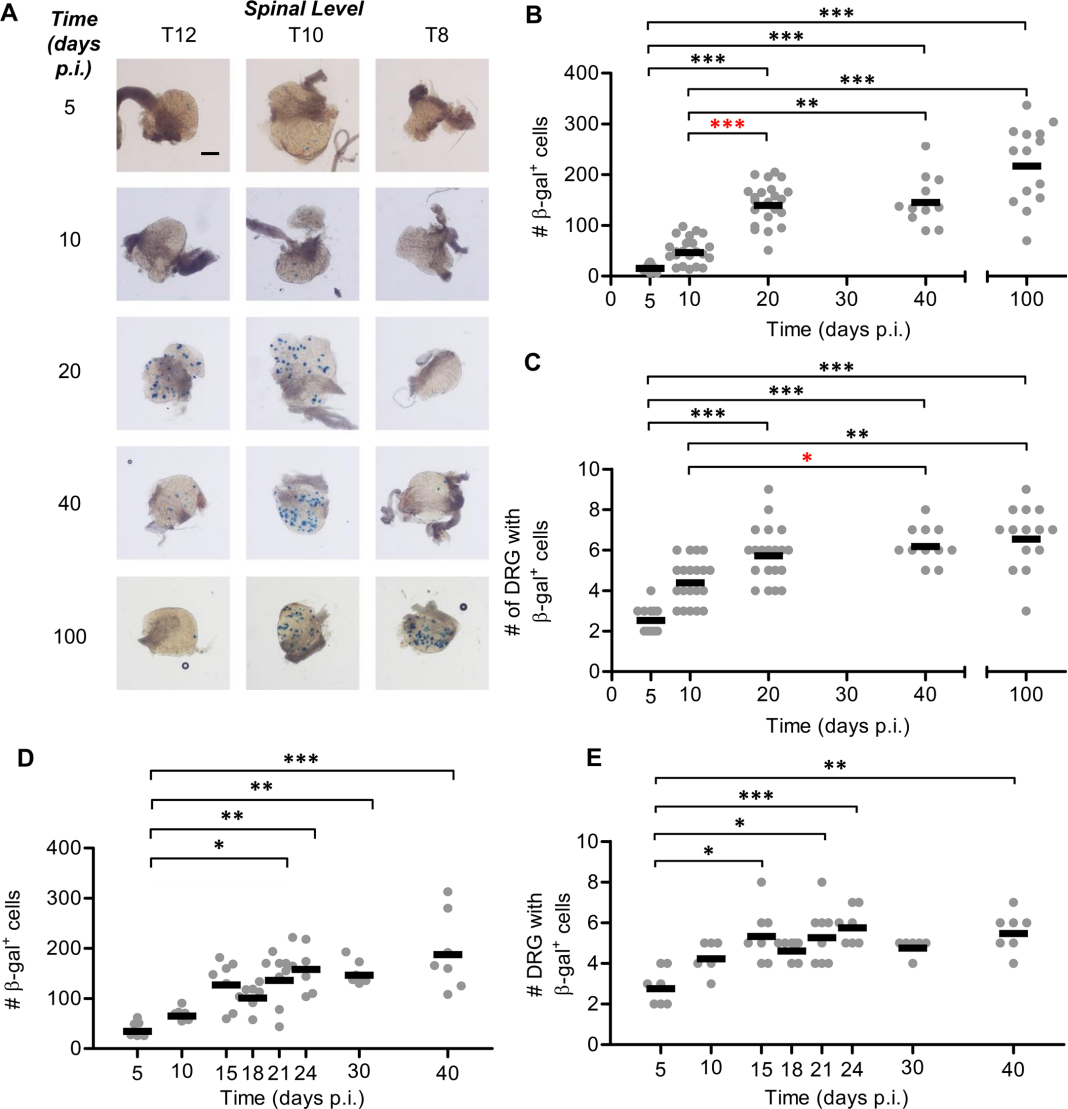
The promoter for ICP47 drives protein expression during the establishment of latency. Groups of ROSA26 mice were infected with HSV-1 pICP47_eGC by tattoo on the flank and were culled 5, 10, 20 and 40, or 10, 20 and 100 days p.i. for determination of the number of β-gal^+^ cells per DRG. (A) Representative photomicrographs of DRG at spinal levels T12, T10 and T8 of a single mouse for each day p.i. taken at 40× magnification (scale bar = 300 μm, as indicated on top left image). (B) The total number of β-gal^+^ cells per mouse and (C) the spread of virus as indicated by the number of DRG containing β-gal^+^ cells was determined. The data were pooled from six independent experiments, with each point representing a single mouse and the bar representing the mean cell count (*n* = 14–22 per day p.i.). (D) The total number of β-gal^+^ cells per mouse and (E) the spread of virus as indicated by the number of DRG containing β-gal^+^ cells confirmed the gradual accumulation of β-gal marked cells during the establishment of latency. The data were pooled from two independent experiments, with each point representing a single mouse and the bar representing the mean cell count (*n* = 6–8 per day p.i.). (*p < 0.05, **p < 0.01, ***p < 0.001; red denotes differences of particular note).

To explore further the relationship between adaptive immunity and viral gene expression through all phases of HSV-1 infection, we examined a virus where Cre was driven by the promoter for ICP47. This promoter is duplicated, being in a repeat region, and the other copy drives ICP22, but for brevity we refer to it here in relation to ICP47 only. ICP47 is of interest to us because it is deployed by HSV as a counter-measure against CD8^+^ T cells [33, 35] and might be predicted to be expressed at any times that it is advantageous for the virus to avoid the consequences of immune recognition. It is also of the immediate early class, like ICP0. When ROSA26R mice were infected with HSV expressing Cre from the ICP47 promoter, the number of marked neurons increased between days 5 and 10 as was now expected (Fig 7A and 7B). Surprisingly, and in contrast to data obtained using the other viruses presented here, an even more striking increase in marking was seen between days 10 and 20. The number of DRG containing at least one marked neuron also increased between days 10 and 20 (Fig 7C). These data suggest that expression of the ICP47 promoter is associated with neuronal survival. Beyond day 40 there appeared to be continued accumulation of marked neurons, but the difference was not statistically significant according to the very conservative (non-parametric) test we have used. We note that a standard ANOVA with several different post-tests for pair-wise comparisons suggest that the rise during latency is significant, but the differing variance and sample sizes across time points in these experiments guided us to use a less powerful test. The unexpected finding of continued marking of neurons during the establishment of latency (days 10 to 20) was then confirmed in an experiment looking at additional times after infection (Fig 7D and 7E). Finally, the remarkable timing of marking by the ICP47 promoter prompted three additional controls. First, we showed that infectious virus was only found in mice infected by this virus during the acute infection (S4 Fig). Second, we monitored eGFP from HSV-1 pICP47_eGC and as expected, frequent eGFP expression was only detected at day 4 (S5A–S5C Fig). Third, we examined the same ICP47 promoter inserted at a different location and using a different fluorescent protein (S5D–S5F Fig) and found a similar pattern of fluorescent marking as with HSV-1 pICP47_eGC. Together these data suggest that the ICP47 promoter used here has the expected pattern of expression.

**Fig 8:**
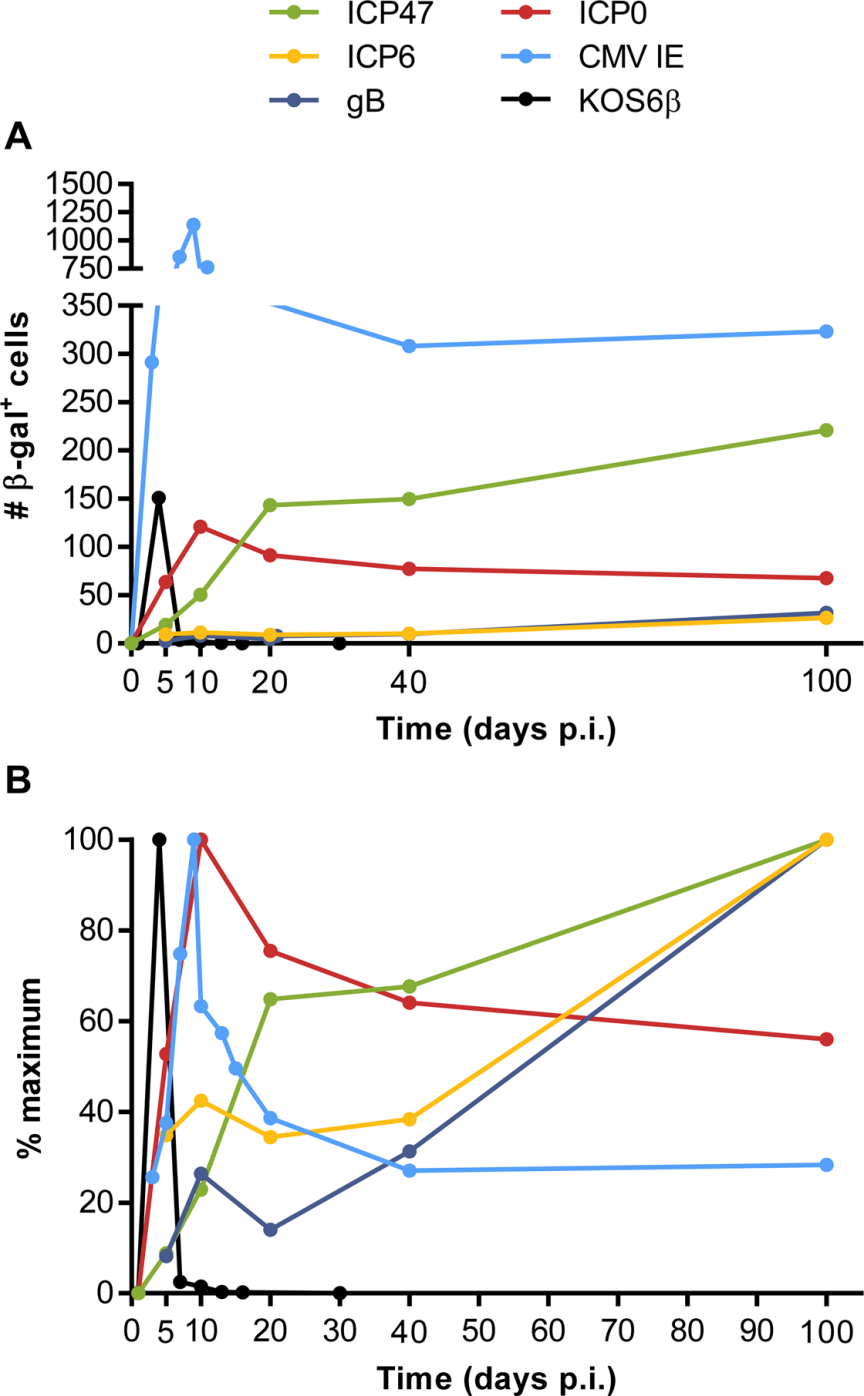
Summary. Data from all experiments where β-gal-marked neurons were counted in DRG after infection of mice with recombinant viruses. The viruses are identified by the gene that was the source of promoter used to drive Cre, or by name (in the case of KOS6β). (A) Total numbers of marked neurons and (B) numbers expressed as a percentage of the maximum counted at the peak time point.

The spread of virus after the peak of the acute infection, coinciding with the frequent expression of a promoter driving an immune evasion gene at this same time is intriguing. We suggest that the time between the acute infection and complete establishment of latency represents an ongoing battle between virus and host. However, the host has clear ascendancy at this time and the remaining viral activity is hard to observe by conventional means. Further, the comparison of results when Cre was under the control of the ICP0 and ICP47 suggests that kinetic class is not a good indicator of likely expression pattern in neurons in vivo. This is not the first time that such a discrepancy has been noted, but is the most striking example to date [55]. Finally we note that number of neurons marked by day 100 when Cre is expressed under the ICP47 promoter approaches that seen after marking by Cre under control of the CMV IE promoter (Fig 8A). As the latter approximates all latently-infected neurons, it would seem likely that given enough time they will all also experience ICP47 expression, further supporting the possibility that this viral function supports neuronal survival.

### Summary and conclusions

Most interpretations of the results presented here require comparisons across multiple viruses and so all the data have been brought together in a summary figure (Fig 8). The first panel highlights the very different levels of marking that can be seen across the viruses and the second highlights the kinetic differences.

The most significant finding remains the detection of protein production driven by lytic gene promoters during latency. The main caveat of this conclusion is that our viruses all have promoters taken out of their natural context and driving a foreign gene. Expression of a viral gene is not only dictated by its promoter, but also by its association with chromatin, location in the genome and the structure of the resulting mRNA. All of these factors need to be considered. Also our promoter sequences were based on the literature, but might not be complete and in the case of ICP47, the promoter had to be modified to remove an origin of replication. However, the position used to insert our constructs is surrounded by lytic genes and our promoters performed as expected *in vitro* and *in vivo* based on eGFP expression. We also note that the promoter for ICP0 failed to generate protein detectable by our system when placed in the same location and this is the second such observation for this promoter in a Cre marking model [29]. While the relatively high level of marking in acute infection with this promoter limits the ability to detect new neurons marked during latency, we would contend that some evidence for increase by day 100 would still be expected (even if it did not reach significance as was the case for the ICP47 promoter, Fig 8B). A final caveat to comparisons across our viruses is that we are unable to account for the possibility that differential expression levels of Cre might lead to different efficiency of loxP recombination.

The interpretation of marking by Cre expressed from gB and ICP6 also bears further discussion. We would interpret the lower early marking by Cre when expressed from the promoters for ICP6 and gB (Fig 8A) to suggest that relatively few neurons that progress beyond early gene expression are able to survive in the acute phase of infection. A corollary of this argument is that because we detect a proportionally greater buildup of neurons marked by these promoters in latency, we are detecting a program of transcription that is distinct from full engagement of the lytic cascade. This, and the finding that full virus spread is achieved by day 10 (Fig 2), suggests again that we are not seeing full reactivation and spread. The most likely interpretations in our view are a limited molecular reactivation [12] or stochastic (or noise in) expression [56] of these genes. In either case the outcome for gB and ICP6 could be detection and response by CD8^+^ T cells [25–27].

In conclusion, these experiments are the most direct evidence to date showing that HSV-1 lytic promoters are capable of generating protein during latency establishment and latency. Further, by using promoters for genes associated with immune recognition, we have linked the observation of CD8^+^ T cell activation in latently-infected DRG with evidence of viral gene expression during latency. Based on these considerations, it is reasonable to speculate that gene expression and detection by the adaptive immune system may be an integral part of the co-evolved host-pathogen relationship that we observe as viral latency.

## Materials and Methods

### Viruses and cell lines

All viruses were grown and titrated on Vero cells (ATCC CCL-81, obtained from the American Type Culture Collection (ATCC),10801 University Boulevard, Manassas, VA 20110 USA). The cycloheximide reversal and acyclovir inhibition assay was performed in Vero SUA cells [57]. Both cell lines were maintained in Minimal Essential Medium (Life Technologies) supplemented with 2 or 10% heated inactivated fetal calf serum (Serana), 5 mM HEPES, 4 mM L-glutamine and 50 μM 2-mercaptoethanol (Life Technologies). All transfections were carried out in 293A cells (from Drs Jon Yewdell and Jack Bennink, NIAID, NIH) using Lipofectamine 2000 (Life Technologies).

HSV-1 KOS.6β [39] was provided by Francis Carbone (University of Melbourne, Australia). HSV-1 pC_eGC and HSV-1 pICP47/Tdtom have been previously described[38].

### Plasmid construction

All sequence references below are to the HSV-1 KOS genome accession JQ673480. Briefly, the plasmids used to construct the recombinant viruses all contained the eGFP/Cre fusion gene (amplified from pIGCN21 [58]) and bovine growth hormone (BGH) polyA termination sequence (amplified from pTracer CMV/bsd; Life Technologies), inserted into the SpeI site of pT U_L_3/U_L_4[38], along with the required promoter sequence. To construct pT pgB_eGC, the U_L_27 (gB) promoter (55985–56282) was amplified from HSV-1 KOS[59], and inserted with eGFP/Cre and the BGH polyA into pT U_L_3/U_L_4. To construct pT pICP6_eGC, the U_L_39 (ICP6) promoter (85920–86166) was amplified from HSV-1 KOS[60], and inserted with eGFP/Cre and the BGH polyA into pT U_L_3/U_L_4. To construct pT pICP47_eGC, the U_S_12 (ICP47) promoter (HSV-1 145998–146497), synthesised by GenScript), with origin of replication (OriS) sequence (HSV-1 145533–145577) removed was inserted with the eGFP/Cre fusion gene and BGH polyA into pT U_L_3/U_L_4. Finally, to construct pT pICP0_eGC, the ICP0 promoter (1271–2238) was amplified from HSV-1 KOS [30], and inserted into pT U_L_3/U_L_4 along with the eGFP/Cre fusion gene and the BGH polyA.

### Construction of recombinant viruses

To construct each of the recombinant viruses HSV-1 pICP47_eGC, HSV-1 pICP6_eGC or HSV-1 pgB_eGC, linearized plasmid DNA from pT pICP47_eGC, pT pICP6_eGC or pT pgB_eGC respectively was cotransfected into Vero cells with HSV-1 KOS genomic DNA. After three days growth at 37°C, 5% CO_2_, plaques containing the desired recombinant virus were identified based on eGFP expression and PCR screening, and four rounds of plaque purification were carried out.

To construct HSV-1 pICP0_eGC, transfection of linearised pT pICP0_eGC DNA with pX330-mC followed by infection with HSV-1 pCmC was carried out as previously described [38]. Desired recombinant virus was identified based on eGFP and mCherry expression, and PCR screening, and four rounds of plaque purification were carried out.

PCR screening and sequencing were used to ensure that the viruses contained the desired modification and were free from parental virus.

### Cycloheximide reversal and acyclovir inhibition assay for promoter characterization

Confluent monolayers of Vero SUA cells (from Prof. Stacey Efstathiou, NIBSC, UK) were pre-treated with 100 μg/mL cycloheximide or 50 μM acyclovir, or left untreated, for one hour at 37°C with 5% CO_2_. The cells were infected at an MOI of 5 either with or without drug as appropriate. After incubation for 1 hour at 37°C with 5% CO_2_, the unabsorbed virus was removed and replaced with fresh media containing the appropriate drug. The cells were then incubated for six h at 37°C, 5% CO_2_, after which time media containing cycloheximide was removed and replaced with 5 μg/mL actinomycin D. The cells were incubated for a further four hours at 37°C, 5% CO_2_. The cells were then harvested, fixed with 1% paraformaldehyde, and the expression of eGFP was assessed using flow cytometry using a LSR-II flow cytometry (BD biosciences). Flow cytometry analysis was performed with the aid of FlowJo 8.7.1 software (Treestar).

### Multiple step growth analysis

Confluent Vero cell monolayers in six well plates were infected with 1 x 10^4^ PFU (MOI 0.01) virus in 1 mL M0. After 1 h at 37°C, virus inocula were removed, the cell monolayer was washed once and 2 mL fresh M2 added. 0 hour p.i. samples were harvested immediately after the addition of fresh media, and further wells harvested at indicated time points into existing media. Virus output was determined by plaque assay on Vero cells.

### Ethics statement

Mice were housed and experiments carried out according to ethical requirements and under approval of the Animal Ethics Committee of the Australian National University (Protocol Numbers A2011.001, A2011.015, and A2014.025). All work complies with the Australian Code for the Care and Use of Animals for Scientific Purposes, 8th edition (2013) and the Australian Capital Territory (ACT) Animal Welfare Act 1992.

### Mice and infections

Female specific pathogen free C57Bl/6 or B6.129S4-Gt(ROSA)26Sor^tm1So^/J (referred to as ROSA26) mice [61] greater than 8 weeks of age were obtained from the Australian Phenomics Facility (Canberra, Australia). Mice were infected using a flank infection model where 1 × 10^8^ PFU/mL virus was introduced into the shaved flank by tattoo under anaesthesia by intraperitoneal injection with Avertin (2, 2, 2-Tribromoethanol) as previously described [38]. The same virus dose and route of infection were used for all experiments.

### Titration of virus from skin and DRG

A 1 cm^2^ portion of skin located over the inoculation site and the DRG found on the ipsilateral side corresponding to spinal levels L1 –T5 were collected 5 days post infection (dpi). Skin or DRG were homogenized in M2. Homogenates were subjected to three cycles of freeze/thawing and infectious virus quantified by plaque assay on Vero cells.

### Histochemical detection of β-gal expression and detection of fluorescence in whole DRG

Mice were culled by C0_2_ asphyxiation and the relevant DRG were removed as soon as possible and fixed in 2% paraformaldehyde/ 0.5% glutaraldehyde for 1 hour on ice. To detect fluorescence in whole DRG, they were then washed with phosphate buffered saline (PBS) before being mounted on glass slides in 50% glycerol in PBS. DRG were visualised and photographed using a Leica DM5500 microscope and DFC365 monochrome camera.

To detect β-gal expression, following fixation DRG were washed 3 times with PBS before incubation for 30 min on ice in permeabilisation solution (0.01% sodium deoxycholate, 2 mM MgCl_2,_ 5 mM potassium ferricyanide, 5 mM potassium ferrocyanide and 0.02% IGEPAL (NP-40) in PBS; Sigma). DRG were then incubated in the permeabilisation solution containing 1 mg/mL X-gal for 16 hours. DRG were washed with PBS again and left in 50% glycerol to clarify overnight[62]. The DRG were then visualised and photographed using an Olympus CKX41 light microscope and Olympus DP20 camera. Large β-gal^+^ cells were assumed to be neurons and their number was determined with the aid of ImageJ software[63].

### Statistical analysis

Statistical comparisons were performed with the aid of Prism software (version 5.01; GraphPad). Differences between groups in all experiments were assessed using a Kruskal Wallis test with Dunn’s post-test for pairwise comparisons. This is a non-parametric test that is conservative, and was chosen because it requires the least number of assumptions to be made about the structure of the data being analyzed. In particular it allows for differences in sample size and variation across populations being compared.

## Supporting Information

S1 Fig Confirmation of kinetic class of HSV-1 promoters. To confirm the expression kinetics of the *eGFP/Cre* fusion gene in the recombinant viruses, Vero cell monolayers were infected in the absence or presence of acyclovir or cycloheximide. Cells were infected with a high MOI (5 PFU/cell) of the indicated virus for 1 hour, before the inoculum was replaced with fresh medium in the presence or absence of the appropriate drug. Cells were incubated for 6 hours at 37°C, 5% CO_2_, before the cycloheximide block was removed and replaced with actinomycin D. Cells were fixed and eGFP expression was assessed by flow cytometry, where the shaded grey area represents eGFP expression in wt HSV-1 infected cells, and the expression of eGFP is shown in blue (A; untreated), red (B; acyclovir treated) or green (C; cycloheximide) treated.
(PDF)

S2 Fig HSV-1 that express *eGFP/cre* from the U_L_3/U_L_4 intergenic region are not impaired for growth relative to wildtype virus. To assess the growth of the recombinant HSV-1 *in vitro* that contain an *eGFP/cre* expression cassette, multiple step growth analysis (MOI 0.01) in Vero cells were performed to compare the growth of the parent HSV-1 virus (shown in black) to either (A) HSV-1 pC_eGC (shown in blue), (B) HSV-1 pICP47_eGC (shown in red), (C) HSV-1 pICP0_eGC (shown in green), (D) HSV-1 pICP6_eGC (shown in yellow) or (E) HSV-1 pgB_eGC (shown in purple). Data are mean±SEM of 3 replicates. To assess the growth of these viruses *in vivo*, groups of 3 or 4 C57Bl/6 mice were infected with 1ˣ10^8^ PFU/mL HSV-1 by tattoo. At 5 days p.i., mice were culled and the amount of infectious virus was determined by standard plaque assay from 10 DRG (spinal levels L1 to T5) or 1 cm^2^ skin located over the site of infection. Circles show results for each mouse and bars represent mean±SEM. (F) Comparison of amount of virus in C57Bl/6 mice infected with HSV-1 KOS (shown in black) or HSV-1 pC_eGC (shown in blue). (G) Comparison of amount of virus in C57Bl/6 mice infected with HSV-1 KOS (shown in black) or HSV-1 pICP0_eGC (shown in green). The means were compared in each tissue by an unpaired *t* test, but in all cases the difference was not statistically significant. (H) Comparison of amount of virus in C57Bl/6 mice infected with HSV-1 KOS (shown in black), HSV-1 pICP47_eGC (shown in red), HSV-1 pICP6_eGC (shown in yellow) or HSV-1 pgB_eGC (shown in purple). The amount of virus in the skin or DRG was compared to that of HSV-1 KOS using an ANOVA with Dunnett’s posttest, but in all cases the differences in means was not statistically significant (*p* > 0.05).
(PDF)

S3 Fig β-gal expression is not detectable in ROSA26 mice latently infected with a *cre* null HSV-1 virus. Groups of 5 ROSA26 mice were infected with either HSV-1 pICP47_eGC (Cre^+^) or HSV-1 pICP47/Tdtom (Cre^-^) and after 20 days the expression of β-gal in their DRG was determined. (A) Representative photomicrographs of DRG at spinal levels T11, T10 and T9 of a single mouse for each virus taken at 40ˣ magnification (scale bar = 300 μm, as indicated on top left image). (B) The total number of β-gal^+^ cells per mouse, with the results of two independent experiments pooled. Each point representing a single mouse and the bar represents the mean cell count (*n* = 10 per virus).
(PDF)

S4 Fig Infectious virus is not detectable during latency in ROSA26 mice infected with HSV-1 pICP47_eGC. Groups of 12 ROSA26 mice were infected with HSV-1 pICP47_eGC, culled at 4, 20 or 40 days p.i. and the quantity of infectious virus within the innervating DRG (spinal levels L1 to T5) was determined. The results of two independent experiments are pooled, with circles showing results for each mouse and bars representing the mean virus titer (*n* = 8 per timepoint).
(PDF)

S5 Fig The promoter for ICP47 drives the expected pattern of conventional fluorescent reporter expression during HSV infection. Groups of C57Bl/6 mice were infected with HSV-1 pICP47_eGC by tattoo on the flank and were culled at various times p.i. and the number of eGFP^+^ cells in each DRG was determined. (A) Representative photomicrographs of DRG at T10 of a single mouse taken at 4 days p.i. and at T11 of another mouse taken at 7 days p.i. at 50× magnification (top; scale bar = 250 μm) and 100× magnification (bottom; scale bar = 100 μm). (B) The total number of eGFP^+^ cells per mouse and (C) the spread of virus as indicated by the number of DRG containing at least one eGFP^+^ cell. Each point represents a single mouse and the bar represents the mean cell count. The results were pooled from two independent experiments (*n* = 8 for each time point) and differences between groups were assessed using a Kruskal Wallis test with Dunn’s posttest for pairwise comparisons (***p < 0.001). Groups of C57Bl/6 mice were infected with HSV-1 pICP47/Tdtom by tattoo on the flank and were culled at various times p.i. and the number of Tdtomato^+^ cells in each DRG was determined. (D) Representative photographs of DRG at T10 of a single mouse taken at 4 or 7 days p.i. at 50× magnification (top; scale bar = 250 μm) and 100× magnification (bottom; scale bar = 100 μm). (E) The total number of Tdtomato^+^ cells per mouse and (F) the spread of virus as indicated by the number of DRG containing at least one Tdtomato^+^ cell. Each point represents a single mouse and the bar represents the mean cell count. The results are pooled from two independent experiments (*n* = 7–8 for each time point) and the differences between the groups were assessed using a Kruskal Wallis test with Dunn’s posttest for pairwise comparisons (*p < 0.05, **p < 0.01, ***p < 0.001).
(PDF)
