# Supplementary material for: Lytic Promoters Express Protein during Herpes Simplex Virus Latency
Source: PLoS Pathog. 2016 Jun 27;12(6):e1005729. doi: 10.1371/journal.ppat.1005729 (PMC4922595; doi:10.1371/journal.ppat.1005729)
Supplement: S1 Fig — To confirm the expression kinetics of the eGFP/Cre fusion gene in the recombinant viruses, Vero cell monolayers were infected in the absence or presence of acyclovir or cycloheximide. Cells were infected with a high MOI (5 PFU/cell) of the indicated virus for 1 hour, before the inoculum was replaced with fresh medium in the presence or absence of the appropriate drug. Cells were incubated for 6 hours at 37°C, 5% CO2, before the cycloheximide block was removed and replaced with actinomycin D. Cells were fixed and eGFP expression was assessed by flow cytometry, where the shaded grey area represents eGFP expression in wt HSV-1 infected cells, and the expression of eGFP is shown in blue (A; untreated), red (B; acyclovir treated) or green (C; cycloheximide) treated. (PDF) [file ppat.1005729.s001.pdf]

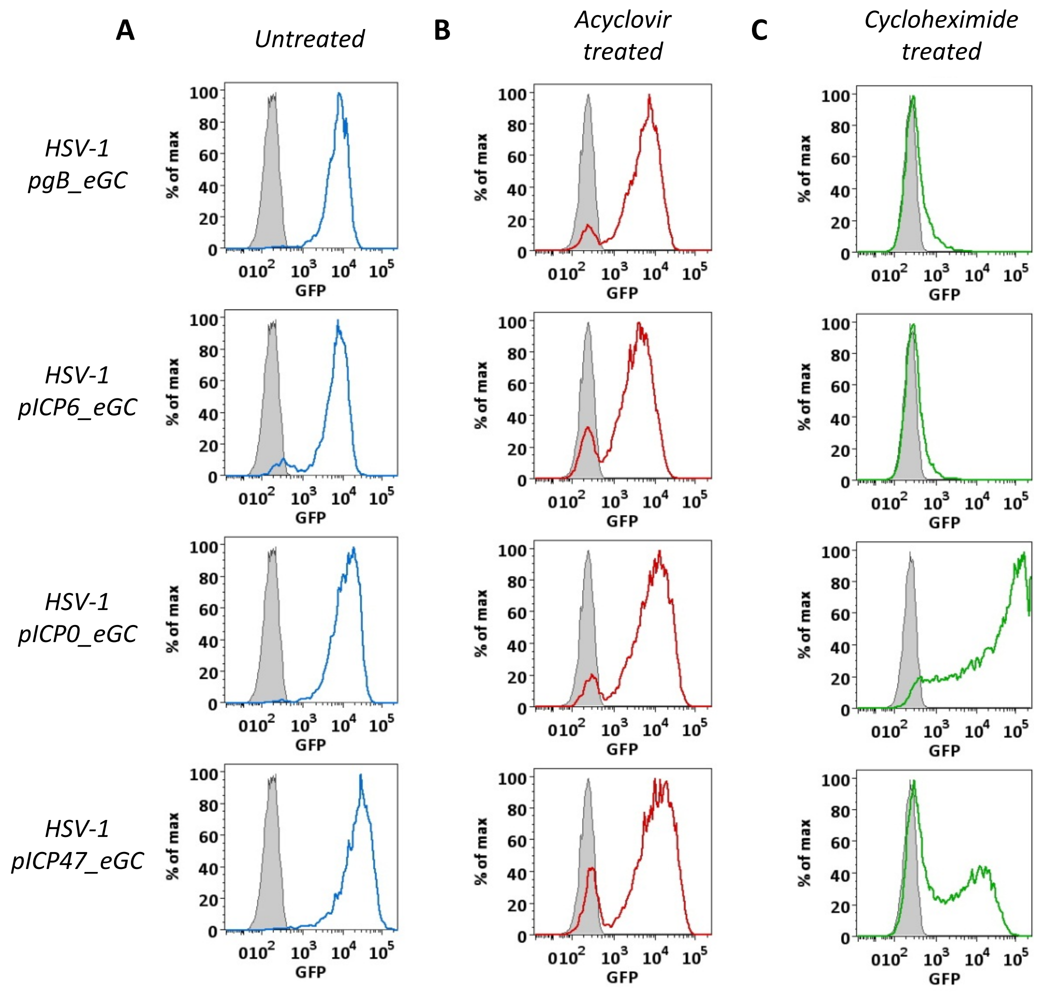

S1 Fig. Confirmation of kinetic class of HSV-1 promoters

To confirm the expression kinetics of the *eGFP/Cre* fusion gene in the recombinant viruses, Vero cell monolayers were infected in the absence or presence of acyclovir or cycloheximide. Cells were infected with a high MOI (5 PFU/cell) of the indicated virus for 1 hour, before the inoculum was replaced with fresh medium in the presence or absence of the appropriate drug. Cells were incubated for 6 hours at 37°C, 5% CO_2, before the cycloheximide block was removed and replaced with actinomycin D. Cells were fixed and eGFP expression was assessed by flow cytometry, where the shaded grey area represents eGFP expression in wt HSV-1 infected cells, and the expression of eGFP is shown in blue (A; untreated), red (B; acyclovir treated) or green (C; cycloheximide) treated.
