# Supplementary material for: Lytic Promoters Express Protein during Herpes Simplex Virus Latency
Source: PLoS Pathog. 2016 Jun 27;12(6):e1005729. doi: 10.1371/journal.ppat.1005729 (PMC4922595; doi:10.1371/journal.ppat.1005729)
Supplement: S2 Fig — To assess the growth of the recombinant HSV-1 in vitro that contain an eGFP/cre expression cassette, multiple step growth analysis (MOI 0.01) in Vero cells were performed to compare the growth of the parent HSV-1 virus (shown in black) to either (A) HSV-1 pC_eGC (shown in blue), (B) HSV-1 pICP47_eGC (shown in red), (C) HSV-1 pICP0_eGC (shown in green), (D) HSV-1 pICP6_eGC (shown in yellow) or (E) HSV-1 pgB_eGC (shown in purple). Data are mean±SEM of 3 replicates. To assess the growth of these viruses in vivo, groups of 3 or 4 C57Bl/6 mice were infected with 1ˣ108 PFU/mL HSV-1 by tattoo. At 5 days p.i., mice were culled and the amount of infectious virus was determined by standard plaque assay from 10 DRG (spinal levels L1 to T5) or 1 cm2 skin located over the site of infection. Circles show results for each mouse and bars represent mean±SEM. (F) Comparison of amount of virus in C57Bl/6 mice infected with HSV-1 KOS (shown in black) or HSV-1 pC_eGC (shown in blue). (G) Comparison of amount of virus in C57Bl/6 mice infected with HSV-1 KOS (shown in black) or HSV-1 pICP0_eGC (shown in green). The means were compared in each tissue by an unpaired t test, but in all cases the difference was not statistically significant. (H) Comparison of amount of virus in C57Bl/6 mice infected with HSV-1 KOS (shown in black), HSV-1 pICP47_eGC (shown in red), HSV-1 pICP6_eGC (shown in yellow) or HSV-1 pgB_eGC (shown in purple). The amount of virus in the skin or DRG was compared to that of HSV-1 KOS using an ANOVA with Dunnett’s posttest, but in all cases the differences in means was not statistically significant (p > 0.05). (PDF) [file ppat.1005729.s002.pdf]

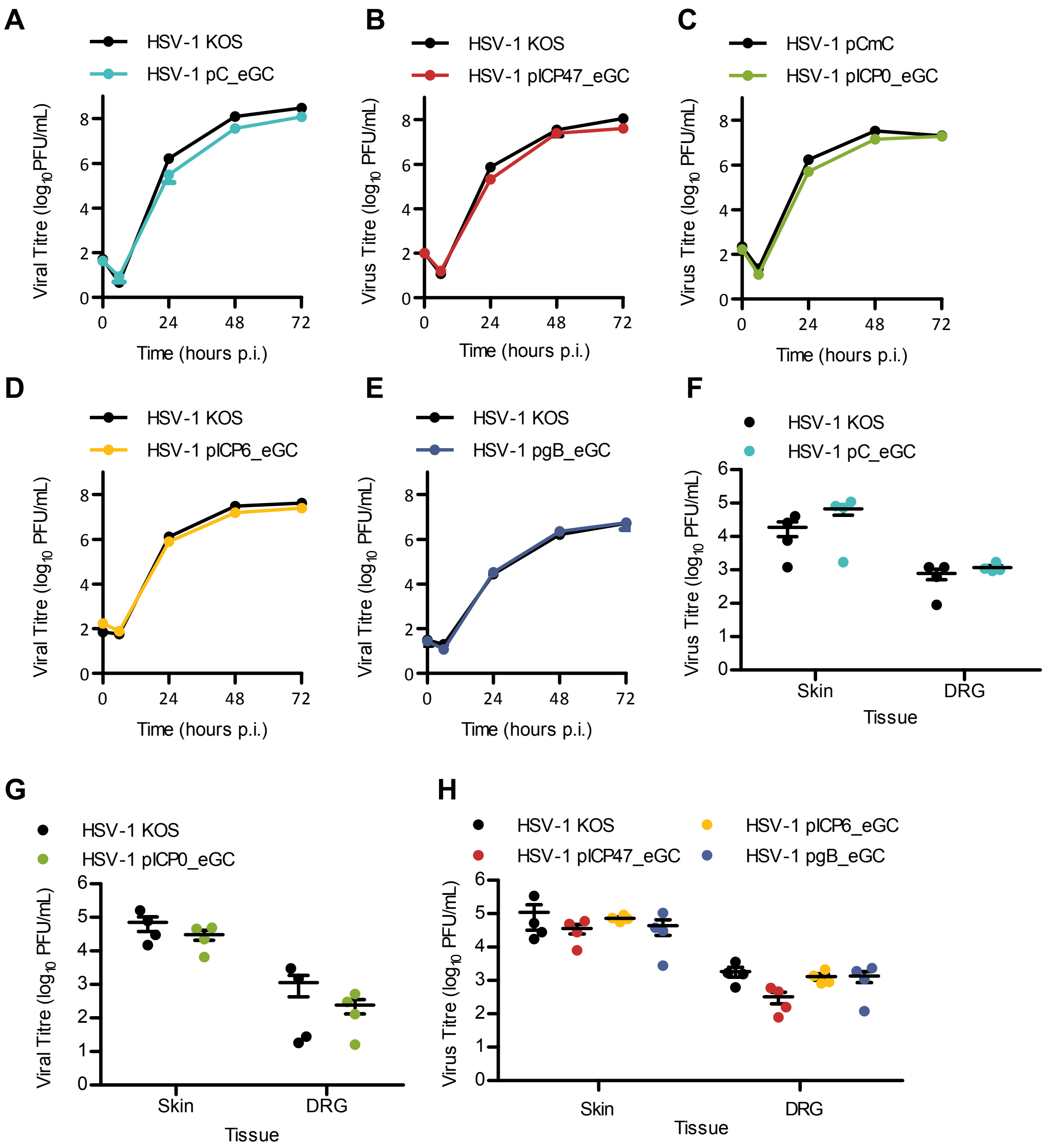

S2 Fig. HSV-1 that express *eGFP/cre* from the U_L3/U_L4 intergenic region are not impaired for growth relative to wildtype virus.

To assess the growth of the recombinant HSV-1 *in vitro* that contain an *eGFP/cre* expression cassette, multiple step growth analysis (MOI 0.01) in Vero cells were performed to compare the growth of the parent HSV-1 virus (shown in black) to either (A) HSV-1 pC_eGC (shown in blue), (B) HSV-1 pICP47_eGC (shown in red), (C) HSV-1 pICP0_eGC (shown in green), (D) HSV-1 pICP6_eGC (shown in yellow) or (E) HSV-1 pgB_eGC (shown in purple). Data are mean±SEM of 3 replicates. To assess the growth of these viruses *in vivo*, groups of 3 or 4 C57Bl/6 mice were infected with 1×10^{8} PFU/mL HSV-1 by tattoo. At 5 days p.i., mice were culled and the amount of infectious virus was determined by standard plaque assay from 10 DRG (spinal levels L1 to T5) or 1 cm^2 skin located over the site of infection. Circles show results for each mouse and bars represent mean±SEM. (F) Comparison of amount of virus in C57Bl/6 mice infected with HSV-1 KOS (shown in black) or HSV-1 pC_eGC (shown in blue). (G) Comparison of amount of virus in C57Bl/6 mice infected with HSV-1 KOS (shown in black) or HSV-1 pICP0_eGC (shown in green). The means were compared in each tissue by an unpaired *t* test, but in all cases the difference was not statistically significant. (H) Comparison of amount of virus in C57Bl/6 mice infected with HSV-1 KOS (shown in black), HSV-1 pICP47_eGC (shown in red), HSV-1 pICP6_eGC (shown in yellow) or HSV-1 pgB_eGC (shown in purple). The amount of virus in the skin or DRG was compared to that of HSV-1 KOS using an ANOVA with Dunnett's posttest, but in all cases the differences in means was not statistically significant ($p > 0.05$).
