# Supplementary material for: Lytic Promoters Express Protein during Herpes Simplex Virus Latency
Source: PLoS Pathog. 2016 Jun 27;12(6):e1005729. doi: 10.1371/journal.ppat.1005729 (PMC4922595; doi:10.1371/journal.ppat.1005729)
Supplement: S3 Fig — Groups of 5 ROSA26 mice were infected with either HSV-1 pICP47_eGC (Cre+) or HSV-1 pICP47/Tdtom (Cre-) and after 20 days the expression of β-gal in their DRG was determined. (A) Representative photomicrographs of DRG at spinal levels T11, T10 and T9 of a single mouse for each virus taken at 40ˣ magnification (scale bar = 300 μm, as indicated on top left image). (B) The total number of β-gal+ cells per mouse, with the results of two independent experiments pooled. Each point representing a single mouse and the bar represents the mean cell count (n = 10 per virus). (PDF) [file ppat.1005729.s003.pdf]

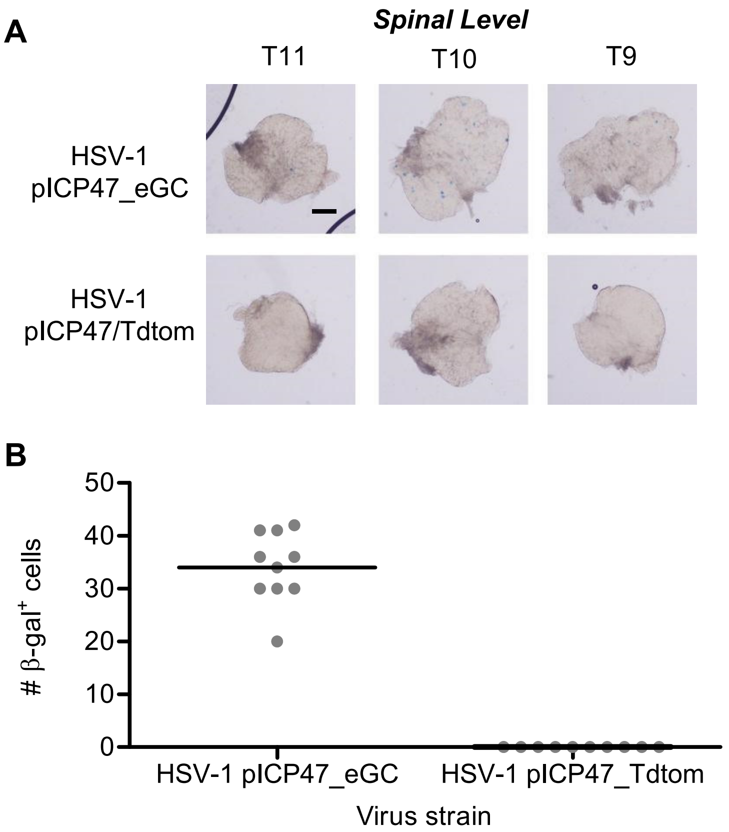

S3 Fig. β-gal expression is not detectable in ROSA26 mice latently infected with a *cre* null HSV-1 virus.

Groups of 5 ROSA26 mice were infected with either HSV-1 pICP47_eGC (Cre^+) or HSV-1 pICP47/Tdtom (Cre^-) and after 20 days the expression of β-gal in their DRG was determined. (A) Representative photomicrographs of DRG at spinal levels T11, T10 and T9 of a single mouse for each virus taken at 40^x magnification (scale bar = 300 μm, as indicated on top left image). (B) The total number of β-gal^+ cells per mouse, with the results of two independent experiments pooled. Each point representing a single mouse and the bar represents the mean cell count (*n* = 10 per virus).
