# Supplementary material for: Lytic Promoters Express Protein during Herpes Simplex Virus Latency
Source: PLoS Pathog. 2016 Jun 27;12(6):e1005729. doi: 10.1371/journal.ppat.1005729 (PMC4922595; doi:10.1371/journal.ppat.1005729)
Supplement: S4 Fig — Groups of 12 ROSA26 mice were infected with HSV-1 pICP47_eGC, culled at 4, 20 or 40 days p.i. and the quantity of infectious virus within the innervating DRG (spinal levels L1 to T5) was determined. The results of two independent experiments are pooled, with circles showing results for each mouse and bars representing the mean virus titer (n = 8 per timepoint). (PDF) [file ppat.1005729.s004.pdf]

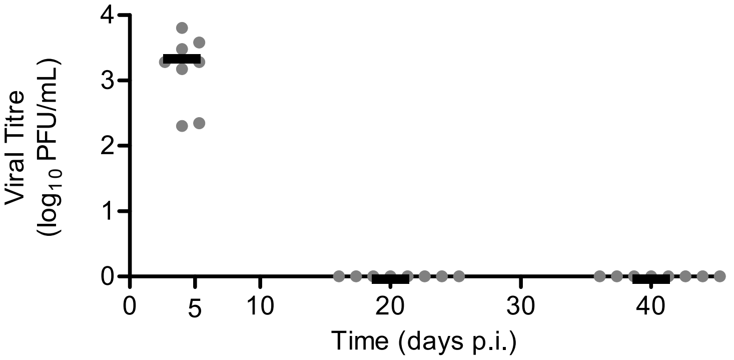

S4 Fig. Infectious virus is not detectable during latency in ROSA26 mice infected with HSV-1 pICP47_eGC.

Groups of 12 ROSA26 mice were infected with HSV-1 pICP47_eGC, culled at 4, 20 or 40 days p.i. and the quantity of infectious virus within the innervating DRG (spinal levels L1 to T5) was determined. The results of two independent experiments are pooled, with circles showing results for each mouse and bars representing the mean virus titre (*n* = 8 per timepoint).
