# Supplementary material for: Lytic Promoters Express Protein during Herpes Simplex Virus Latency
Source: PLoS Pathog. 2016 Jun 27;12(6):e1005729. doi: 10.1371/journal.ppat.1005729 (PMC4922595; doi:10.1371/journal.ppat.1005729)
Supplement: S5 Fig — Groups of C57Bl/6 mice were infected with HSV-1 pICP47_eGC by tattoo on the flank and were culled at various times p.i. and the number of eGFP+ cells in each DRG was determined. (A) Representative photomicrographs of DRG at T10 of a single mouse taken at 4 days p.i. and at T11 of another mouse taken at 7 days p.i. at 50× magnification (top; scale bar = 250 μm) and 100× magnification (bottom; scale bar = 100 μm). (B) The total number of eGFP+ cells per mouse and (C) the spread of virus as indicated by the number of DRG containing at least one eGFP+ cell. Each point represents a single mouse and the bar represents the mean cell count. The results were pooled from two independent experiments (n = 8 for each time point) and differences between groups were assessed using a Kruskal Wallis test with Dunn’s posttest for pairwise comparisons (***p < 0.001). Groups of C57Bl/6 mice were infected with HSV-1 pICP47/Tdtom by tattoo on the flank and were culled at various times p.i. and the number of Tdtomato+ cells in each DRG was determined. (D) Representative photographs of DRG at T10 of a single mouse taken at 4 or 7 days p.i. at 50× magnification (top; scale bar = 250 μm) and 100× magnification (bottom; scale bar = 100 μm). (E) The total number of Tdtomato+ cells per mouse and (F) the spread of virus as indicated by the number of DRG containing at least one Tdtomato+ cell. Each point represents a single mouse and the bar represents the mean cell count. The results are pooled from two independent experiments (n = 7–8 for each time point) and the differences between the groups were assessed using a Kruskal Wallis test with Dunn’s posttest for pairwise comparisons (*p < 0.05, **p < 0.01, ***p < 0.001). (PDF) [file ppat.1005729.s005.pdf]

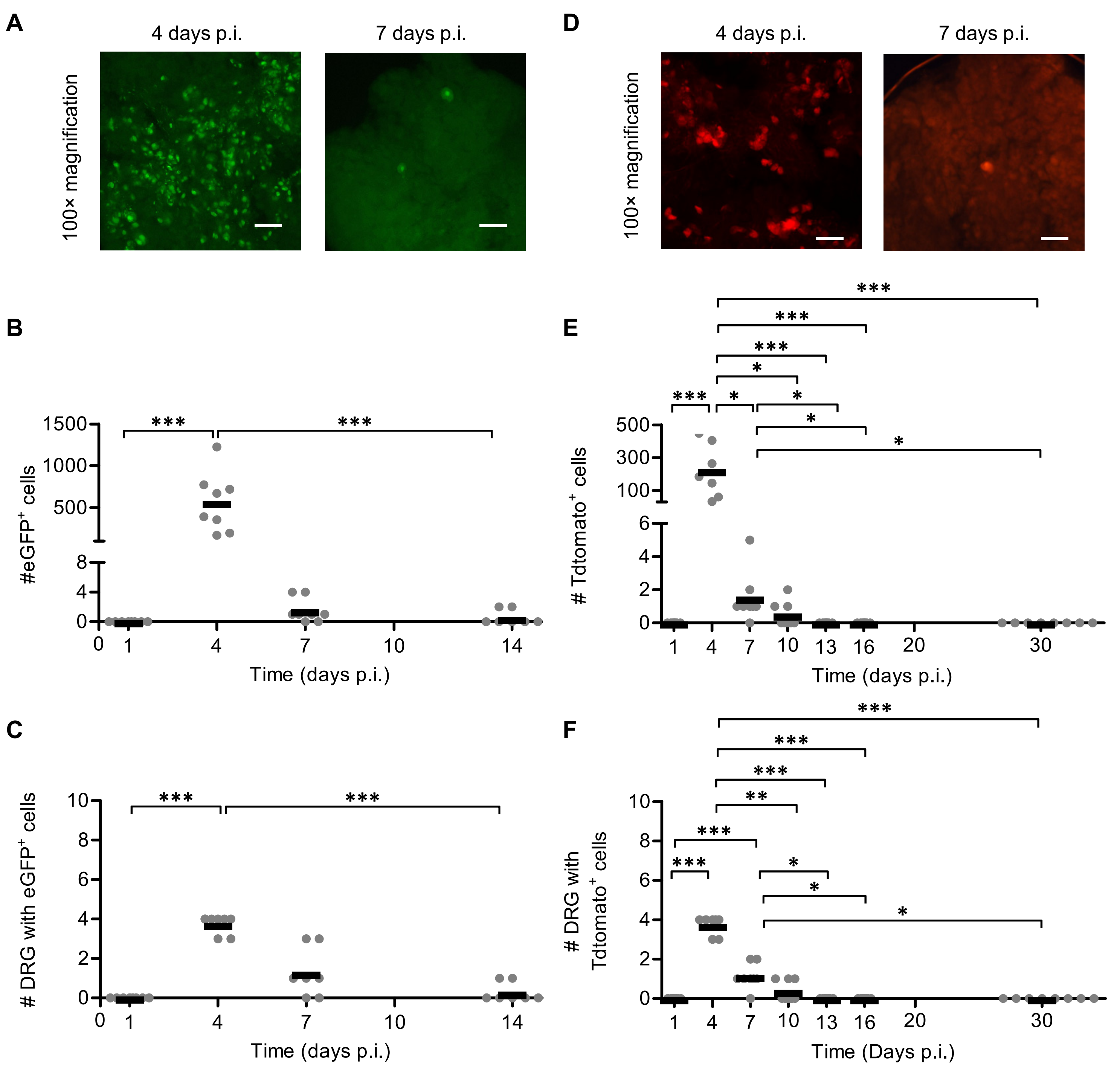

S5 Fig. The promoter for ICP47 drives the expected pattern of conventional fluorescent reporter expression during HSV infection.

Groups of C57Bl/6 mice were infected with HSV-1 pICP47_eGC by tattoo on the flank and were culled at various times p.i. and the number of eGFP$^+$ cells in each DRG was determined. (A) Representative photomicrographs of DRG at T10 of a single mouse taken at 4 days p.i. and at T11 of another mouse taken at 7 days p.i. at 50× magnification (top; scale bar = 250 µm) and 100× magnification (bottom; scale bar = 100 µm). (B) The total number of eGFP$^+$ cells per mouse and (C) the spread of virus as indicated by the number of DRG containing at least one eGFP$^+$ cell. Each point represents a single mouse and the bar represents the mean cell count. The results were pooled from two independent experiments (n = 8 for each time point) and differences between groups were assessed using a Kruskal Wallis test with Dunn's posttest for pairwise comparisons (***p < 0.001). Groups of C57Bl/6 mice were infected with HSV-1 pICP47/Tdtom by tattoo on the flank and were culled at various times p.i. and the number of Tdtomato$^+$ cells in each DRG was determined. (D) Representative photographs of DRG at T10 of a single mouse taken at 4 or 7 days p.i. at 50× magnification (top; scale bar = 250 µm) and 100× magnification (bottom; scale bar = 100 µm). (E) The total number of Tdtomato$^+$ cells per mouse and (F) the spread of virus as indicated by the number of DRG containing at least one Tdtomato$^+$ cell. Each point represents a single mouse and the bar represents the mean cell count. The results are pooled from two independent experiments (n = 7 – 8 for each time point) and the differences between the groups were assessed using a Kruskal Wallis test with Dunn's posttest for pairwise comparisons (*p < 0.05, **p < 0.01, ***p < 0.001).
